# The approach of physiotherapists in the management of patients with persistent pain and comorbid anxiety/depression: are there any differences between male and female professionals?

**DOI:** 10.1192/j.eurpsy.2024.1671

**Published:** 2024-08-27

**Authors:** G. Nicolini, M. Chiesa, M. Buoli

**Affiliations:** ^1^Department of Mental Health, Department of Biomedical and Clinical Sciences Luigi Sacco, Luigi Sacco Hospital, University of Milan; ^2^Department of Psychiatry, University of Milan; ^3^Department of Neurosciences and Mental Health, Fondazione IRCCS Ca’ Granda Ospedale Maggiore Policlinico, Milan, Italy

## Abstract

**Introduction:**

Chronic pain is a prevalent condition that is frequently complicated by concomitant mood and anxiety disorders. Very preliminary data indicate that female physiotherapists could have a better attitude towards psychiatric disorders.

**Objectives:**

Purpose of the present article is to identify eventual differences in the management of patients with chronic pain and anxiety/mood disorders depending on the physiotherapists’ gender.

**Methods:**

An ad-hoc questionnaire was developed and sent to physiotherapists by e-mail. The two group identified by gender were compared by unpaired sample t tests for continuous variables and χ2 tests for qualitative ones. A binary logistic regression was then performed with factors resulted statistically significant at univariate analyses as independent variables and gender as dependent one.

**Results:**

Female physiotherapists (compared to male ones) resulted to be more confident in the prosecution of physiotherapy by patients with Generalized Anxiety Disorder (GAD)comorbidity (t=2.46, p=0.01) and by patients who had received a visit with a mental health professional (t=2.79, p=0.01). Furthermore, female physiotherapists versus male ones believed that pharmacotherapy was less associated with motor side effects (t=2.90, p<0.01) and more frequently recognized the importance of a training to identify affective disorders (t=2.65, p=0.01) and the need of more education in mental health (t=2.85, p=0.01). The binary logistic regression model confirmed that female professionals (compared to male ones) were less likely to work as freelance in private institutions (p=0.015) and were more confident in the prosecution of physiotherapy by patients with GAD comorbidity (p=0.05).

**Conclusions:**

Female compared to male physiotherapists resulted to be more comfortable with patients affected by mental conditions and to be more aware of the need of training on mental health. Implementation of mental health education for male physiotherapists is probably necessary and further studies are needed to confirm the results of the present study.

**Disclosure of Interest:**

None Declared

